# An examination of whether associations exist between maternal and neonatal 25OHD and infant size and adiposity at birth, 6–9 months and 2–2.5 years of age – a longitudinal observational study from the ROLO study

**DOI:** 10.1186/s40795-017-0184-9

**Published:** 2017-07-17

**Authors:** Mary K. Horan, Jean M. Donnelly, Malachi J. McKenna, Brenda Crosbie, Mark T. Kilbane, Fionnuala M. McAuliffe

**Affiliations:** 1 UCD Obstetrics and Gynaecology, School of Medicine and Medical Science, University College Dublin, National Maternity Hospital, Dublin 2, Ireland; 2 Department of Endocrinology, St. Vincent’s University Hospital, Dublin 4, Ireland; 3 Metabolism Laboratory, St Vincent’s University Hospital, Dublin 4, Ireland

**Keywords:** Fetal programming, Maternal vitamin D, Offspring adiposity, Childhood obesity, Maternal nutrition, ROLO study, J0101

## Abstract

**Background:**

Vitamin D status in pregnancy and offspring bone health effects are well established, yet limited knowledge exists on the effect of maternal vitamin D status on offspring size/adiposity. This study examines the association of early (13 weeks), late (28 weeks) pregnancy and neonatal (umbilical) 25-hydroxyvitamin D (25OHD) on offspring size/adiposity.

**Methods:**

This analysis included mother-infant pairs from the ROLO study at birth (*n* = 292), 6–9 months (*n* = 160) and 2–2.5 years (*n* = 287) postpartum.

**Results:**

Using Institute of Medicine 2011 Report criteria, 30% of women in early pregnancy and 38% in late pregnancy were at risk of vitamin D deficiency (25OHD < 30 nmol/L). Birthweight was negatively associated with early-pregnancy 25OHD (*p* = 0.004) and neonatal 25OHD (*p* < 0.001). Birth length was not associated with 25OHD. Neonatal measures of overall adiposity were negatively associated with neonatal 25OHD (*p* = 0.001, and *p* = <0.001 respectively). At 2–2.5 years there was a negative association between weight-for-age z-score and early-pregnancy 25OHD (*p* < 0.041).

**Conclusions:**

Maternal and neonatal 25OHD were negatively associated with offspring size/adiposity at birth and offspring weight-for-age at 2–2.5 years. Results may not reflect a general population replete in vitamin D, due to high prevalence of macrosomia and high risk of deficiency in this cohort. Improvement of pregnancy vitamin D status remains a public health concern.

**Trial registration:**

Current Controlled Trials ISRCTN54392969. 22/04/2009 retrospectively registered.

**Electronic supplementary material:**

The online version of this article (doi:10.1186/s40795-017-0184-9) contains supplementary material, which is available to authorized users.

## Background

The role of vitamin D in bone health is well established, while evidence of its role in extra-skeletal health continues to accumulate [1, 2]. Vitamin D is derived from oral intake and from skin production by UVB radiation. Vitamin D derived from both sources is then hydroxylated in the liver to make 25-hydroxyvitamin D (25OHD) and again in the kidney to the most active form 1, 25-dihydroxyvitamin D. In Ireland the main contribution to dietary vitamin D is from meat, followed to a lesser extent by fish, spreads, eggs and milk and yogurts [3]. The different sources of vitamin D increase the complexity of research in the area. Production due to sun exposure depends on multiple factors including latitude and UltraViolent B (UVB) radiation levels, skin colour, sunscreen use, covering up and practices such as purdah [4]. Similarly, oral intake depends on vitamin D levels in plant and animal foods consumed, food fortification, supplement intake, and practices such as vegetarianism and veganism [5, 6].

During pregnancy, maternal 25OHD tends to decline, but maternal production of 1, 25-dihydroxyvitamin D increases because there is up regulation of renal enzymes in order to facilitate the doubling of maternal intestinal calcium absorption during pregnancy [7–9]. Vitamin D is not required by the fetus for calcium absorption because calcium is actively transported across the placenta; vitamin D becomes essential for calcium absorption only postpartum [8]. Offspring vitamin D status at birth may be determined by measuring umbilical cord 25OHD, which correlates with maternal 25OHD [8, 10, 11]. The placenta contains 1 α-hydroxylase which converts 25OHD to the active form but its contribution to maternal calcium balance is uncertain [8, 10, 12].

There is evidence that maternal and fetal vitamin D status or season of birth may be associated with offspring adiposity [13–17]. However, some studies have reported no such associations between maternal 25OHD and offspring health [18–23]. The aim of this study was to examine the association between maternal and neonatal 25OHD levels and infant size and adiposity at birth, 6–9 months and 2–2.5 years of age in a cohort from the ROLO (Randomised cOntrol trial of LOw glycaemic index diet versus no dietary intervention to prevent recurrence of fetal macrosomia) study [24]. In a previous analysis of a subgroup of the ROLO study, late maternal 25OHD above the cohort median was associated with significantly greater neonatal length than late maternal 25OHD below the median [25].

## Methods

Mother and infant pairs from the ROLO study were included in this analysis at birth (*n* = 292), at 6–9 months postpartum (*n* = 160), and at 2–2.5 years postpartum (*n* = 287). Of these, 159 were assessed at all three time-points (birth, 6 months and 2 years of age) and had a measurement of maternal 250HD in early pregnancy; 166 were assessed at all three time-points and had a measurement of maternal 25OHD in late pregnancy; and, 120 were assessed at all three time-points and had a measurement of neonatal 25OHD. The ROLO study was a randomised control trial of low glycaemic index diet in pregnancy to reduce macrosomia. 800 euglycaemic secundigravid women who had previously given birth to a macrosomic baby (> 4 kg), and were therefore at increased risk of delivering another macrosomic infant [26], were randomised to receive low glycaemic index (GI) dietary advice or usual antenatal care, which did not include dietary advice. Detailed methodology and results of the ROLO study have been published previously [24, 27]. In brief, the primary outcome was a reduction in birthweight and the secondary outcomes were a reduction in gestational weight gain and glucose intolerance. The primary outcome was not achieved while the secondary outcomes were both achieved. Low GI dietary advice was given at week 14 of pregnancy and demographic, well-being and lifestyle questionnaires were returned by 28 weeks gestation. 3-day food diaries were completed during each trimester of pregnancy and used to determine the glycaemic index and glycaemic load of the women’s diets, both of which were significantly reduced following the intervention [24, 28]. This study was conducted according to the guidelines laid down in the Declaration of Helsinki with institutional ethics approval from ethics committee of the National Maternity Hospital, Ireland, for the original study, and further ethical approval from the same ethics committee for the follow-up study up to 2 years of age was obtained. Informed written maternal consent was obtained at each time-point of the study. Trial Registration with Current Controlled Trials www.controlled-trials.com ISRCTN54392969. Informed written maternal consent was obtained from all participants both during pregnancy and at the 2 year follow-up appointment.

### Inclusion and exclusion criteria

Participants were secundigravida women who had previously given birth to a macrosomic infant (> 4 kg). They were required to have sufficient literacy and English language fluency to understand the intervention and complete questionnaires. Participants were required to have healthy, singleton pregnancies with no intrauterine growth abnormalities. All participants were invited to return for a follow-up appointment with their infant both at 6 months postpartum and at 2 years postpartum. Infants were eligible for inclusion until they reached 9 months of age for the first postnatal follow-up and 2 years 6 months of age for the second postnatal follow-up. Participants who returned for follow-up at 2 years postpartum were chosen for 25OHD analysis of stored blood samples and their anthropometric measurements at birth and at 6 months were also examined, where available. It was of interest to examine long-term associations between 25OHD in pregnancy and offspring adiposity; 2 years was the latest follow-up age available so a sample of convenience was chosen.

### Maternal demographics, lifestyle and infant feeding practices

Of the 800 participants of the ROLO study, 25OHD was measured in 300 participants. Of these, 292 anthropometric measurements were performed for 292 infants at birth, 160 at 6–9 months of age, and 287 at 2–2.5 years of age. Questions on lifestyle habits were taken from SLAN (Survey of Lifestyle, Attitudes and Nutrition in Ireland) including questions on physical activity, smoking, education and food label reading [29].

### Maternal and infant anthropometry

Maternal weight, height and mid-upper arm circumference were measured at the first antenatal consultation and body mass index was calculated. Maternal weight and mid-upper arm circumference were also measured at 6–9 months and 2–2.5 years postpartum and body mass index (BMI) calculated. Neonatal weight and length was measured for all infants at birth while mid-upper arm, abdominal, hip and thigh circumference, and biceps, triceps, subscapular and thigh skinfold thickness measurements were initiated later in the study and therefore complete anthropometric data was available for only 122 neonates in this cohort. Birthweight centile was calculated using Gestation Network’s Bulk Calculator version 6.2.3, UK. Centiles were corrected for maternal weight, height, parity and ethnicity and infant gestational age at delivery and gender [30]. The same anthropometric measurements were also taken at 6–9 months and 2–2.5 years of age. WHO growth standards were used to convert these measurements to z-scores which adjusts for infant age at exam (in days) and gender and report standard deviations away from the mean [31, 32]. Six-nine month and 2–2.5 year olds were also classified according to WHO BMI-for-age z-score cut-offs as wasted or severely wasted (BMI-for-age z-score < −2), normal (BMI-for-age z-score − 2 to 1), at risk of overweight (BMI-for-age z-score 1.01–2), overweight (BMI-for-age z-score 2.01–3) and obese (BMI-for age z-score > 3). Waist:hip, waist:length and subscapular skinfold:triceps skinfold ratios as well as sum of triceps and subscapular skinfold thicknesses and sum of all skinfold thicknesses were calculated as markers of infant adiposity.

### Maternal supplement use, season of pregnancy, ethnicity and infant feeding practices

Information on maternal supplement use was collected during pregnancy as a binary yes/no question. Specific data on maternal vitamin D supplementation or sunlight exposure were not collected. The season of pregnancy was classified into summer or winter according to the month in which the baby was delivered; delivery from June to November was classified as a summer pregnancy while delivery from December to May was classified as a winter pregnancy [25]. Maternal ethnicity was recorded. At 6–9 months and 2–2.5 years postpartum mothers completed a questionnaire about feeding practices, such as whether or not infant had ever been breastfed, the duration of breastfeeding, the age at which solids and other drinks, including infant formula, were introduced.

### 25OHD measurement

Maternal blood samples were taken in early pregnancy at the first antenatal appointment at 13 weeks gestation and in late pregnancy at 28 weeks gestation. Cord blood samples were taken at delivery. These samples were centrifuged at 3000 rpm for 10 min and serum sub-aliquoted and stored at minus 80 °C until analysis. Samples were excluded from analysis if they were haemolysed (early pregnancy *n* = 0, late pregnancy *n* = 2, cord *n* = 27). Serum total 25OHD concentrations were measured using the Elecsys vitamin D total automated competitive binding protein assay (Roche Diagnostics GmbH, Mannheim, Germany). This method employs a recombinant vitamin D binding protein as capture protein and is standardised against liquid chromatography tandem mass spectroscopy, which in turn has been standardised to The National Institute for Standards and Technology standard. Our laboratory has a proficiency certificate issued consecutively over the last 10 years, acknowledging the fact that our assay meets the performance standards set by the world’s leading vitamin D quality assurance scheme (DEQAS). The Roche 25OHD method does not discriminate the 3-epimer of 25OHD. Current evidence suggests that the 3-epimer of 25OHD is present in all human serum and not just neonates less than 1 year. Concentrations of 3-epimer of 25OHD accounted for 11% of maternal, 14% of cord and 25% of infant 25OHD in the paper by Bailey et al. [33] (below). One could argue therefore that 3-epimer of 25OHD levels in mother and cord blood samples are not significantly different from each other, in terms of affecting data analysis.

The coefficients of variation (CV) for the 25OHD assay determined at assay validation were: interassay CV = 8.9% at a concentration of 49.5 nmol/L, 3.7% at 103 nmol/L; intra-assay CV = 2.9% at a concentration of 49.5 nmol/L, 1.4% at 103 nmol/L. We participate in the Vitamin D External Quality Assessment Scheme [34]. We interpreted maternal 25OHD according to North American Institute of Medicine (IOM) Dietary Reference Intake (DRI) committee, which specified a 25OHD corresponding to risk of deficiency at <30 nmol/L, to the estimated average requirement (EAR) at 40 nmol/L, and to the recommended daily allowance at 50 nmol/L [2, 35].

### Statistical analysis

The study represents a sub-analysis of a primary randomized control trial, ROLO, which was designed with very different hypotheses and objectives to those that form the basis of this report. This study was not planned a priori and represents a sample of convenience for a sub-analysis. Results are presented as mean (standard deviation) or as number (percent). Statistical analyses entailed simple and multiple linear regression modelling using a forced enter approach. Known confounders were controlled for in these models as follows: (1) at birth, these variables were season of pregnancy, maternal education, maternal BMI in early pregnancy, any supplement use during pregnancy, maternal smoking, offspring gender and gestational age; and (2) at 6–9 months and 2–2.5 years, these variables were season of pregnancy, maternal education, any supplement use during pregnancy, maternal smoking, offspring gender and age at assessment (weeks) and finally duration of breastfeeding. Models predictive of infant anthropometric measurements, for which z-scores were available, were not adjusted for infant age and gender as these factors are taken into account when converting to z-scores. Control and intervention groups were analysed together but study group was controlled for in all final models. Multiple linear regression resulted in a best and final model, and models that were statistically significant (*p* < 0.05) were reported. For 6 month and 2 year olds, they were also grouped into the dichotomies “not overweight or at risk of overweight” and “overweight or obese” using World Health Organisation (WHO) BMI-for age cut-off [32]. Due to small numbers in the wasted and overweight and obese groups; the association of maternal and neonatal 25OHD with these dichotomies was then further analysed using t-tests. Statistical analysis was performed using IBM SPSS for Windows, version 20.0 (Armonk, NY).

## Results

### Background demographics

Maternal demographics and offspring anthropometry are displayed in Table 1. There was no difference in background demographics between the control and intervention groups similar to the original ROLO study [24]. Mothers in this cohort were relatively well educated with 56.1% having completed third level education. Maternal age at delivery was 33.0 ± 3.9 years. Maternal BMI was 26.07 ± 4.40 kg/m^2^ and 1.7% of mothers reported smoking during pregnancy. Although the intervention group of this cohort had significantly lower post-intervention glycaemic index in trimester 2 and 3 similarly to the original ROLO study (56.4 ± 3.9 vs. 57.7 ± 3.4, *p* = 0.010 and 55.7 ± 3.8 vs. 57.7 ± 3.9, *p* < 0.001 respectively) there was no significant difference in gestational weight gain between the two groups (*p* = 0.570) unlike in the original study [24]. According to the World Health Organisation (WHO) BMI-for-age classification [32] at 6–9 months, 7 infants (4.4%) were classified as wasted, 133 (83.1%) as normal, 14 (8.8%) as at risk of overweight, 6 (3.8%), and as overweight or obese (0%). At 2–2.5 years of age, according to the WHO BMI-for-age classification, 2 infants (0.7%) were classified as wasted, 207 (71.9%) as normal, 51 (17.7%) as at risk of overweight, 25 (8.7%) as overweight and 3 (1.0%) as obese. Mothers who participated at 2–2.5 years were compared with those who did not and were found to have a higher education level, were less likely to be smokers and had a lower BMI at baseline than those who did not attend (Additional file 1: Table S1). These factors were controlled for in the analysis*.*

**Table 1:** Background characteristics of the ROLO cohort for whom vitamin D levels were measured

Variable	Mean	SD
Early pregnancy 25OHD (nmol/L)	41.8	19.5
Late pregnancy 25OHD (nmol/L)	40.0	21.4
Neonatal vitamin 25OHD (nmol/L)	44.6	26.1
Mother age at delivery (years)	33.0	3.94
Mother height (cm)	165	15.1
Mother weight booking (kg)	72.1	12.6
Mother MUAC at booking (cm)	29.2	3.15
Mother BMI at booking (kg/m^2^)	26.1	4.40
Gestational weight gain (kg)	13.6	4.64
Length of gestation (days)	283	7.46
Birthweight (kg)	4.06	0.46
Birthweight centile	72.5	25.0
Birth length (cm)	52.9	2.25
Birth head circumference (cm)	35.8	1.66
Birth abdominal circumference (cm)	33.6	1.93
Birth chest circumference (cm)	35.6	2.27
Birth hip circumference (cm)	33.7	2.31
Birth mid upper arm circumference (cm)	12.6	1.16
Birth thigh circumference (cm)	16.2	1.68
Birth subscapular SFT (mm)	6.95	1.58
Birth biceps SFT (mm)	6.72	1.56
Birth triceps SFT (mm)	6.97	1.53
Birth thigh SFT (mm)	7.81	1.78
Birth waist:hip circumference ratio	1.00	0.06
Birth waist circumference:length ratio	0.64	0.04
Birth sum of all SFT (mm)	28.5	5.37
Birth sum of triceps SFT and subscapular SFT (mm)	13.9	2.77
Birth subscapular SFT:triceps SFT ratio	1.02	0.20
Age at 6–9 month assessment (weeks)	28.2	6.69
6–9 month weight-for-age z-score	0.74	1.00
6–9 month length-for-age z-score	1.30	1.12
6–9 month BMI-for-age z-score	0.02	1.04
Age at 2–2.5 year assessment (weeks)	109	9.86
2–2.5 year weight-for-age z-score	0.69	1.00
2–2.5 year length-for-age z-score	0.90	4.87
2–2.5 year BMI-for-age z-score	0.40	1.28
Duration of Breastfeeding (weeks)	13.2	23.6

*SFT* skinfold thickness, *MUAC* mid-upper arm circumference

### Maternal supplement use, season of pregnancy, ethnicity and infant feeding practices

Maternal ethnicity was mainly “white Irish” (92.8%) with only 0.3% of women classified as Filipino or South East Asian, 0.3% as Indian and 0.3% as Chinese, while 6.2% were “white other”. The majority of women (56.6%) were taking some vitamin or mineral supplement during pregnancy, although type of supplement was not recorded. The season of pregnancy was fairly evenly spread as 55.1% of women were classified as having winter pregnancies and 44.9% summer pregnancies. 46.2% of women reported ever breastfeeding the participant infant, while 26.4% reported never breastfeeding and 27.4% did not provide data on breastfeeding. The duration of breastfeeding in the overall cohort was 13.2 ± 23.6 weeks although the duration of breastfeeding for women who reported ever breastfeeding was higher at 28.5 ± 27.7 weeks. Again, there was no difference between the control and intervention groups for any of these variables.

### 25OHD measurements

There was no difference in 25OHD between the control and intervention groups in early or late pregnancy or neonatal blood samples (*p* = 0.568, *p* = 0.074 and *p* = 0.264 respectively). Mean (SD) 25OHD was 41.8 (19.5) nmol/L in early pregnancy, 39.9 (21.4) nmol/L in late pregnancy, and 44.6 (26.2) nmol/L in neonatal cord blood. 25OHD in early and late pregnancy were both positively correlated with neonatal 25OHD (*r* = 0.676, *p* < 0.001 and *r* = 0.761, *p* < 0.001 respectively). When 25OHD concentrations were classified according to IOM guidelines [2], 30.2% of women were <30 nmol/L, 39.5% were between 30 and 50 nmol/L, and 30.2% were >50 nmol/L in early pregnancy. Of those with 25OHD <30 nmol/L in early pregnancy, 47.8% were members of the intervention group. The corresponding frequencies of women in each 25OHD category in late pregnancy were 37.7%, 34.2% and 28.1% respectively, and of those with 25OHD <30 nmol/L, 46.4% were members of the intervention group. There was no association between maternal BMI in early pregnancy and 25OHD in early (*p* = 0.271) or late (*p* = 0.137) pregnancy nor was there any association with early or late pregnancy 25OHD when the WHO BMI classification was used to categorise maternal BMI (*p* = 0.811, *p* = 0.456 respectively). Finally, there was no association observed between vitamin D categories in early or late pregnancy and baseline or 28 weeks gestation maternal glucose levels, nor with development of gestational diabetes. Summer pregnancies (baby delivered between June and November) were associated with significantly higher 25OHD than winter pregnancies in late gestation (44.1 ± 23.8 vs. 36.6 ± 18.7 nmol/L, *p* = 0.003) and in neonatal samples (56.0 ± 30.7 vs. 38.2 ± 19.7 nmol/L, *p* < 0.001), but the opposite was seen in early pregnancy (38.3 ± 19.8 vs. 46.5 ± 18.3 nmol/L, *p* = 0.001). Supplement intake was not associated with absolute levels of maternal or neonatal 25OHD in the overall cohort. However, when the cohort was separated by season, supplement intake was associated with significantly higher 25OHD in late pregnancy during winter (40.0 ± 21.1 vs. 33.0 ± 14.0 nmol/L, *p* = 0.025).

### Association between early and late maternal 25OHD and neonatal 25OHD and offspring size and adiposity

#### Birth anthropometry

Single linear regression analysis showed many significant associations between neonatal anthropometry and early and late pregnancy and neonatal 25OHD (Table 2), but after adjustment for many confounders in the multiple linear regression models there were fewer significant associations (Table 3). After adjustment for confounders, birthweight was negatively associated with early pregnancy and neonatal 25OHD (β = −4.158, *p* = 0.004 and β = −5.919, *P* < 0.001, respectively). Birth length was also negatively associated with early, late pregnancy 25OHD and with neonatal 25OHD; but when birthweight was controlled for in these models, this association lost significance at all time-points (*p* = 0.123, *p* = 0.117 and *p* = 0.244, respectively). Neonatal 25OHD was negatively associated with the following at birth: subscapular skinfold thickness (β = −0.024, *p* < 0.001), sum of all skinfold thicknesses, a measure of overall adiposity, (β = −0.079, *p* = 0.001), and with the sum of the triceps and subscapular skinfold thickness (β = −0.043, *p* < 0.001). Ponderal index at birth was not associated with early or late pregnancy or neonatal 25OHD.

**Table 2:** Simple linear regression showing associations between 25OHD (independent variable) and offspring anthropometrics (dependent variables)

Variable	β	SE	P	F	R^2^
Early pregnancy 25OHD (nmol/L)					
Birthweight (g)	−3.85	1.37	0.005	7.88	0.023
Birthweight centile	−0.25	0.08	0.001	11.3	0.036
Birth length (cm)	−0.01	0.01	0.001	2.72	0.007
Birth head circumference (cm)	−0.01	0.01	0.013	6.28	0.037
6 month head circumference for age z-score	−0.01	0.01	0.021	5.44	0.028
2 year weight for age z score	−0.01	0.00	0.019	5.54	0.016
Late pregnancy 25OHD (nmol/L)					
Birthweight (g)	−1.26	1.27	0.321	0.99	0.000
Birthweight centile	−0.11	0.07	0.126	2.36	0.005
Birth length (cm)	−0.01	0.01	0.057	3.66	0.011
6 month subscapular SFT:triceps SFT ratio	−0.002	0.00	0.040	4.27	0.022
2 year waist:hip ratio	−0.001	0.00	0.007	7.52	0.027
Neonatal 25OHD (nmol/L)					
Birthweight (g)	−4.46	1.18	<0.001	14.2	0.054
Birthweight centile	−0.24	0.06	<0.001	15.2	0.060
Birth length (cm)	−0.02	0.01	0.020	5.52	0.023
Birth abdominal circumference (cm)	−0.02	0.01	0.016	6.05	0.044
Birth thigh circumference (cm)	−0.01	0.01	0.029	4.92	0.035
Birth subscapular SFT (mm)	−0.02	0.01	<0.001	13.7	0.123
Birth biceps SFT (mm)	−0.01	0.01	0.033	4.68	0.039
Birth sum of subscapular and triceps SFT (mm)	−0.03	0.01	0.002	9.97	0.091
Birth subscapular SFT:triceps SFT ratio	−0.002	0.00	0.050	3.94	0.032
Birth sum of SFT (mm)	−0.06	0.02	0.005	8.35	0.076
6 month waist:length ratio	0.00	0.00	0.014	6.19	0.041

*SFT* skinfold thickness, *R* ^*2*^ adjusted coefficient of determination

**Table 3:** Associations between early and late pregnancy and cord 25OHD and offspring size and adiposity- adjusted analysis

Variable	B	SEB	P	R^2^	F	p
Early pregnancy 25OHD (nmol/L)						
Birthweight (g)	−4.16	1.42	0.004	0.20	7.623	<0.001
Birth length (cm)	−0.02	0.01	0.023	0.20	6.639	<0.001
^a^Birth length (cm)	−0.01	0.01	0.123	0.29	9.512	<0.001
2–2.5 year weight for age z score	−0.11	0.00	<0.001	0.12	4.291	<0.001
Late pregnancy 25OHD (nmol/L)						
Birthweight (g)	−2.51	1.34	0.063	0.18	6.729	<0.001
Birth length (cm)	−0.02	0.01	0.028	0.15	5.142	<0.001
^a^Birth length (cm)	−0.01	0.01	0.117	0.29	9.650	<0.001
Neonatal 25OHD (nmol/L)						
Birthweight(g)	−5.92	1.20	<0.001	0.29	10.198	<0.001
Birth length (cm)	−0.02	0.01	0.006	0.20	5.180	<0.001
^a^Birth length (cm)	−0.01	0.01	0.244	0.32	9.074	<0.001
Birth subscapular SFT (mm)	−0.02	0.01	<0.001	0.24	3.534	0.001
Birth sum of all SFT (mm)	−0.08	0.02	0.001	0.12	2.093	0.043
Birth sum of subscapular and triceps SFT (mm)	−0.04	0.01	<0.001	0.16	2.501	0.016

*SFT* skinfold thickness, *R* ^*2*^ adjusted coefficient of multiple determination. Multiple linear regression models for dependent variables at birth were adjusted for season of pregnancy, maternal education, maternal BMI in early pregnancy, any supplement use during pregnancy, maternal smoking, offspring gender and gestational age. ^a^The association between birth length and 25OHD also controlled for birthweight. Multiple linear regression models for dependent variables at 6–9 months and 2–2.5 years were adjusted for season of pregnancy, maternal education, any supplement use during pregnancy, maternal smoking, offspring gender and age at assessment (weeks), and duration of breastfeeding

#### Six-nine month anthropometry

There were no statistically significant associations observed at 6–9 months of age when confounders were controlled for using multiple linear regression. BMI-for-age dichotomies “not overweight or at risk of overweight” vs. “at risk of overweight or overweight or obese” were not associated with early or late pregnancy 25OHD (*p* = 0.170 and *p* = 0.937, respectively) or with neonatal 25OHD (*p* = 0.463).

#### Two-two and a half year anthropometry

The only significant association remaining at 2–2.5 years once confounders were controlled for using multiple linear regression was a negative association between early pregnancy 25OHD and 2–2.5 year old weight-for-age z-score (β = −0.11, *p* < 0.001) (Table 3). BMI-for-age dichotomies “not overweight or at risk of overweight” versus “at risk of overweight or overweight or obese” were not associated with early or late pregnancy 25OHD (*p* = 0.250 and *p* = 0.599 respectively) or with neonatal 25OHD (*p* = 0.326).

## Discussion

The average 25OHD in our maternal cohort, both in early and late gestation, is nearly equivalent to the IOM EAR for healthy population at 40 nmol/L [2, 36] but 30% in early pregnancy and 37% in late pregnancy were at risk of vitamin D deficiency (25OHD <30 nmol/L). These estimates of risk of vitamin D deficiency are substantially higher than prevalence estimate of 7% in healthy Irish adults in the National Adult Nutrition Survey [6]. While we have noted a marked improvement in vitamin D status in our studies over the past three decades, there are still at-risk groups in Ireland such as pregnant women especially during wintertime [25, 37–41].

We found that birthweight was negatively associated with early pregnancy and neonatal 25OHD. Birth length was negatively associated with early and late pregnancy and neonatal 25OHD; however when birthweight was controlled for in these models, this association lost significance at all time-points. Neonatal sum of all skinfolds and sum of subscapular and triceps skinfolds, both measures of overall adiposity, were negatively associated with neonatal 25OHD. There was no association between early or late pregnancy 25OHD or neonatal 25OHD and 6–9 month old anthropometry. At 2–2.5 years of age the only association observed was a negative association between weight-for-age z-score and early pregnancy 25OHD.

Our finding of negative associations between early pregnancy 25OHD and neonatal 25OHD and birthweight is contrary to the findings of several other studies at birth. A recent study by Eckhardt et al. found that maternal 25OHD <30 nmol/L compared to maternal 25OHD ≥30 nmol/L at 20 weeks gestation was associated with lower weight-for-age z-score and BMI-for-age z-score at birth [17]. A study by Leffelaar et al. did not find any association between maternal 25OHD and birthweight once confounders were controlled for, but did find that maternal 25OHD <30 nmol/L compared to 25OHD >50 nmol/L was associated with greater risk of small for gestational age [42]. While another study by Morley et al. found no association between birthweight and early or late pregnancy 25OHD [43]. An older study by Phillips and Young similarly found that birthweight was not associated with month or season of birth although 25OHD was not measured [16]. The contrary nature of our findings of a negative association between maternal 25OHD in early pregnancy and offspring birthweight to those of other studies may be due, in part, to the nature of our cohort, in which there was a high prevalence of macrosomia. Our finding that late pregnancy 25OHD was not significantly associated with birthweight is similar to the study by Gale et al. of a cohort from Southampton which found no association between maternal 25OHD in late pregnancy and birthweight, and to another study by Sayers and Tobias which found that maternal UVB exposure in late pregnancy was not significantly associated with birthweight [23, 44].

Birth length was negatively associated with early and late pregnancy 25OHD and neonatal 25OHD; however, when birthweight was controlled for in these models, to determine if this association was simply a function of birthweight, this association lost significance at all time-points. This finding is similar to a study by Gale et al. found no association between late pregnancy 25OHD and birth length [23]. A study by Morales et al. of early pregnancy 25OHD likewise found no association [15]. Previous studies have also reported positive associations between birth length and maternal 25OHD levels in pregnancy [17] and with maternal UVB exposure [43, 44]. Fewer studies have examined the longer-term associations of maternal or neonatal 25OHD and offspring length. In this cohort, no association was observed between neonatal 25OHD or maternal 25OHD in early or late pregnancy and offspring length at 6–9 months or 2–2.5 years of age which is similar to the results of other longitudinal studies examining these associations at various ages in childhood [15, 23, 45]. Leffelaar et al. [42] also examined offspring height, up to 1 year of age and found that infants of mothers who were vitamin D deficient in early pregnancy were significantly shorter than infants of mothers who were not deficient at 1 month of age. Whereas by 1 year of age this relationship had reversed and the length of infants whose mothers were vitamin D deficient was significantly greater than those of mother without deficiency.

Neonatal sum of all skinfolds and sum of subscapular and triceps skinfolds, both measures of overall adiposity, were negatively associated with neonatal 25OHD in this cohort contrary to results of a recent study by Godang et al. where neonatal 25OHD was positively associated with neonatal fat mass measured by dual electron x-ray absorptiometry (DXA) [14]. Another study, also using DXA, found that late pregnancy 25OHD was positively associated with 3 week old fat mass, which the authors classified as birth adiposity, although neonatal 25OHD was not examined [13]. Finally, a study by Eckhardt et al. found that BMI-for-age z-score at birth was positively associated with maternal 25OHD at 20 weeks gestation [17].

In terms of offspring adiposity in later childhood, at 2–2.5 years old a negative association between weight-for-age z-score and early pregnancy 25OHD was observed in this cohort. Other studies that have examined the association between maternal or neonatal 25OHD and offspring adiposity in later life have reported conflicting results. Gale et al. found no association between late pregnancy 25OHD and offspring fat mass measured using DXA at 9 years of age [23]. While Crozier et al. found that late pregnancy 25OHD was negatively associated with offspring fat mass, also measured using DXA, at 6 years but not at 4 years of age [13]. Leffelaar et al. observed significantly greater offspring weight at 6 and 9 months of age in children whose mothers were deficient rather than sufficient in vitamin D in early pregnancy, however offspring adiposity was not examined [42]. A study of early pregnancy found that 25OHD <50 nmol/L was associated with increased BMI-for-age z-score and risk of overweight at 1 year of age but not at 4 years of age [15]. Whereas, Eckhardt et al. found that BMI-for-age z-score and weight-for-age z-score at 4 months and 1 year of age were not significantly associated with maternal vitamin D deficiency at 20 weeks gestation [17]. A study of 9.9 year old offspring found that maternal UVB exposure in late pregnancy was positively associated with offspring fat mass and weight [44]. An earlier study found that obesity at age 59–73 years was positively associated with winter pregnancies in men with a trend towards the same association in women [16].

This study had some limitations. Detailed data on maternal vitamin D supplement use and maternal sun exposure was not available, but we had data on season of pregnancy and actual measured 25OHD. In addition, as mentioned, complete anthropometric measurements were not available for all neonates although we had weight, length and head circumference for the full cohort which allowed examination of the association between maternal 25OHD and basic measures of neonatal size for the full cohort, with more in depth body composition analysis possible in a subgroup. Finally, data was not available on offspring diet at 2–2.5 years, precluding adjustment for the effect of dietary intake on size and adiposity. However, we did have data on infant feeding practices and duration of breastfeeding, which was controlled for in all analyses. Some variables controlled for in the multiple regression models may have been on the causal pathway such as: season, supplement intake or maternal BMI. However, these variables equally may have been confounders in their own right: season may affect birthweight independently of sunlight exposure [46]; supplement intake may have affected birthweight through supplementation with micronutrients other than vitamin D; and, maternal adiposity may lead to low 25OHD leading to altered birthweight. Regards the latter point, we did not find an association between maternal BMI and vitamin D status. We deemed it appropriate to control for any possible confounders while recognising the limitations of this strategy. There is no uniformity about the definition of season with respect to analysis of 25OHD. Despite improvement in vitamin D in Ireland over the past 40 years, a marked seasonality in 25OHD is still observed [47]. We chose summer/winter definition in order to align with our previous ROLO study analysis [25]. The results of our study may not be generalisable other cohorts due to relatively homogenous ethnicity, high education level and high prevalence of macrosomia and these factors, particularly the risk of macrosomia, which may explain the contradictory results when compared with other studies of maternal 25OHD and offspring size. Unfortunately, selection bias is an issue with all longitudinal studies due to loss to follow-up over time and it was an issue in this study too with mothers who returned at 2–2.5 years post-partum having a higher level of education, lower BMI in early pregnancy and lower smoking rate. However, these factors were controlled for where possible and, while further research is necessary, this study adds important data to the sparse literature on the association between maternal and neonatal 25OHD and offspring size and adiposity at birth and in childhood, and is unique in examining this association using early and late pregnancy and neonatal 25OHD.**5.**

## Conclusions

Maternal and neonatal 25OHD were negatively associated with offspring size and adiposity at birth, and with offspring weight-for-age at 2–2.5 years in a cohort with high prevalence of macrosomia in whom risk of vitamin D deficiency was high. No association was observed with offspring anthropometry at 6–9 months of age. Further research is necessary to determine whether an optimum 25OHD level exists in pregnancy for promotion of healthy offspring size and adiposity. Meanwhile, improvement of vitamin D status in pregnancy remains a public health concern.

## Additional file

Additional file 1: Table S1. Comparison of participants at 2–2.5 years post-partum (who also had vitamin D levels from pregnancy available) and non-responders. (DOCX 15 kb)

## Abbreviations

25OHD: 25-Hydroxyvitamin D
GI: Glycaemic index
UV: Ultraviolet
WHO: World Health Organisation
