# Supplementary material for: An examination of whether associations exist between maternal and neonatal 25OHD and infant size and adiposity at birth, 6–9 months and 2–2.5 years of age – a longitudinal observational study from the ROLO study
Source: BMC Nutr. 2017 Jul 17;3:62. doi: 10.1186/s40795-017-0184-9 (PMC7050699; doi:10.1186/s40795-017-0184-9)
Supplement: Supplementary file 1 — Comparison of participants at 2–2.5 years post-partum (who also had vitamin D levels from pregnancy available) and non-responders. (DOCX 15 kb) [file 40795_2017_184_MOESM1_ESM.docx]

|  | Participants | | Non-responders | |  |
| --- | --- | --- | --- | --- | --- |
|  | n | Mean±SD | n | Mean±SD | p |
| Mother age (years) | 265 | 32.95±3.93 | 440 | 32.47±4.37 | 0.142 |
| Moderate physical activity (hours/week) | 160 | 3.19±2.01 | 206 | 3.31±2.29 | 0.591 |
| Birthweight centile | 277 | 72.48±24.96 | 432 | 72.02±25.62 | 0.894 |
| Maternal BMI baseline (kg/m^2^) | 294 | 26.08±4.43 | 483 | 27.07±5.24 | 0.007 |
| Gestational weight gain (kg) | 156 | 13.64±4.64 | 210 | 12.97±4.72 | 0.180 |
|  | Participants (n=287) | | Non-responders (n=513) | |  |
|  | n (%) | | n (%) | |  |
| Maternal smokers | 5 (1.7)  150 (52.3)  161 (56.1)  152 (53.0) | | 26 (5.1)  236 (46.0)  188 (36.6)  215 (41.9) | | 0.011 |
| Intervention group |  |  |  |  | 0.430 |
| Achieved 3^rd^ level education |  |  |  |  | 0.017 |
| Maternal supplement use in pregnancy |  |  |  |  | 0.756 |

Table S1. Comparison of participants at 2-2.5 years post-partum (who also had vitamin D levels from pregnancy available) and non-responders

Independent sample t-tests and chi-squared tests were used in this analysis. P<0.05 was considered significant
